# An Acid‐Oxidising Solution Containing Hypochlorous Acid Reduces *Staphylococcus aureus* and Improves Bacterial Diversity in Epidermolysis Bullosa Wounds

**DOI:** 10.1111/exd.70147

**Published:** 2025-08-13

**Authors:** Xiaolong A. Zhou, Michael B. Burns, Ziyou Ren, Elise Stagaman, Stefan J. Green, Lok Yiu Ashley Wu, Lynna Yang, Stephanie Rangel, Lydia Rabbaa, Amy S. Paller

**Affiliations:** ^1^ Department of Dermatology Northwestern University, Feinberg School of Medicine Chicago Illinois USA; ^2^ Department of Biology Loyola University Chicago Chicago Illinois USA; ^3^ Genomics and Microbiome Core Facility Rush University Medical Center Chicago Illinois USA

**Keywords:** acid‐oxidising solution, epidermolysis bullosa, hypochlorous acid, microbiome, *Staphylococcus aureus*, wounds

## Abstract

Epidermolysis bullosa (EB) is a group of rare genetic skin disorders characterised by skin fragility and chronic, painful wounds that are highly susceptible to bacterial infection, particularly by *Staphylococcus aureus* (SA). This study evaluated the efficacy of an acid‐oxidising solution containing hypochlorous acid (HOCl) in reducing SA colonisation, promoting wound healing, and restoring a healthier microbiome in EB wounds. In a 12‐week open‐label pilot study, 15 EB patients applied the HOCl‐based spray (APR‐TD011) daily to chronic wounds for 8 weeks, with full‐length 16S rRNA sequencing of wound swabs performed before, during, and after treatment. At baseline, 87% of patients were culture‐positive for SA, and sequencing revealed that SA had the highest relative abundance (34%), followed by *Acinetobacter guillouiae* and *Pseudomonas poae* . SA relative abundance decreased precipitously by Weeks 4 (to 11%) and 8 (primary endpoint; to 10%, *p* < 0.01), and this effect persisted at 4 weeks post‐treatment (Week 12; to 9.7%), including for methicillin‐resistant SA. Concurrently, bacterial diversity increased, and wound sizes diminished in correlation with reduced SA levels (*r* = 0.64). Younger patients exhibited greater SA reduction trends. The treatment was well‐tolerated, with minimal adverse effects and high patient satisfaction. This study underscores the role of microbial dysbiosis in EB wounds and highlights HOCl‐based solutions as a promising therapy to mitigate pathogenic burden and enhance wound healing.

## 1 Introduction

Epidermolysis bullosa (EB) is a group of rare inherited skin disorders characterised by mechanobullous skin fragility, leading to painful and often pruritic chronic and recurrent wounds that frequently become infected with *Staphylococcus* (*S*.) *aureus* (SA). EB simplex (EBS), junctional EB (JEB), and dystrophic EB (DEB) are primary subtypes, each with different pathogenesis, localisation of cutaneous cleavage, and range of severity. Generalised forms of JEB and DEB are associated with considerable disease morbidity and complications [1]. Sepsis is a leading cause of mortality, and chronic EB wounds contribute to poor growth, chronic anaemia, hypoproteinemia, and, particularly with DEB, an increased risk of developing locally aggressive and metastatic squamous cell carcinoma [2, 3]. EB has no cure, and a crucial element of patient management involves proper and timely wound care. While small prior studies have examined the skin microbiome of EB wounds and unaffected skin [4, 5, 6], little is known about the effect of wound care on resident bacterial communities and clinical improvement. Determining this relationship is key to elucidating the relevance of dysbiosis to EB and optimising clinical practices.

Based on cultures, 93% of EB wounds are colonised with *Staphylococcus* species (86% SA, with ~50% methicillin‐resistant SA [MRSA]). Other dominant taxa from cultures include *Streptococcus* species (22%–43%) and *Pseudomonas (P.) aeruginosa* (37%) [7, 8]. These pathogens likely access wounds because of the impaired epidermal barrier and innate immunity, driving a pro‐inflammatory state to exacerbate wound healing impairment and provoke itch [1, 4, 5, 6]. Decreased bacterial diversity and increased SA burden serve as biomarkers for poor wound healing [4], as SA abundance correlates with EB disease severity and acute and chronic wound burden [5]. One study implicated *S. epidermidis a*s the predominant taxon in EB wounds, more so than SA and *P. aeruginosa* [4]. These previous studies utilised 16S rRNA gene amplicon sequencing, targeting either the V1‐V2 or V3‐V4 regions, with limitations regarding species‐level resolution, emphasising the need for full‐length 16S rRNA sequencing to better determine relative abundances of *Staphylococcus* species.

While 70% of EB patients use topical antimicrobials to reduce the burden of a high bacterial load, current wound approaches fail to prevent and eliminate EB wound colonisation [8, 9]. Assessing how specific topical antimicrobial agents, and ideally non‐antibiotic antimicrobial agents, prompt microbiome shifts in EB wounds is key to developing novel strategies for treating EB‐related infections. APR‐TD011 is a commercially‐available acid‐oxidising solution (AOS, Applied Pharma Research, Balerna, Switzerland) containing hypochlorous acid (HOCl 0.0038%, pH 2.5–3.0, > 95% of free chorine species), studied for diabetic and venous stasis ulcers but never EB wounds. Its combination of HOCl, low pH, and high redox potential reduces growth of bacteria and/or biofilm, promotes wound healing, and suppresses the pro‐inflammatory NF‐kB inflammatory pathway and metal metalloproteinases, while preserving cell and tissue viability [10, 11, 12, 13, 14]. Moreover, the product kills both methicillin‐sensitive and methicillin‐resistant SA (MSSA), as well as *P. aeruginosa* , in bactericidal assays [11, 15]. We performed a pilot study of the EB microbiome, the impact of hypochlorous acid on the EB skin microbiota, and the effect on the size and pain of EB wounds of daily use of APR‐TD011 spray for 8 weeks.

## 2 Materials and Methods

### 2.1 Study Design

This 12‐week, single‐centre, open‐label, single‐arm pilot study of 15 EB patients was conducted February 2023 and March 2024 at the Ann & Robert H. Lurie Children's Hospital of Chicago (IRB #2022–5523). Written informed consent was obtained from adult subjects and parents/guardians if < 18 years, with assent from subjects ≥ 12 years old. Inclusion criteria for EB patients were ≥ 6 months of age with ≥ 1 target wound present for ≥ 1 week that cultured positive for SA. Patients using oral antibiotics, topical antibiotics, or other antimicrobials near the target site < 4 weeks of the initial visit were excluded. If an infection developed that required antibiotics, only data points before infection were included.

Patients (or parents/guardians) administered the spray daily for 8 weeks on the selected wound by first gently patting the wound with sterile gauze to remove drainage and then liberally spraying the product on the wound area. After air drying for 2 min, the wound was blotted with a clean gauze, sprayed a second time, and then dressed with the patient's existing non‐adherent dressings. Patient demographics, medical history, and skincare and diet routines were collected. Clinical photographs, wound size measurements, patient‐reported pain scores and product tolerance, and skin culture and microbiome swabs (in triplicate) were obtained from the designated target wound immediately before treatment initiation (Week 0) and at Weeks 4, 8, and 12, the latter 4 weeks after stopping the spray.

Pain during wound dressing changes was assessed once a month using the Wong‐Baker FACES scale of 0 = “no hurt” to 10 = “hurts worst”. Local tolerability was assessed immediately after spraying using a 5‐point Likert scale for both itching and burning/stinging (0 = none; 1 = slight; 2 = mild, 3 = moderate; 4 = severe). Patients with baseline itch at the wound were asked to semi‐quantify any increase in (or lack of) itch from the spray. Patient or parent satisfaction with the product was assessed based on quick drying, feeling good on the skin, preference over other products used in the past (e.g., emollients), and ease of application. Control environmental air swabs were obtained from each visit to assess contamination. Swab specimens were placed immediately in sterile cryovials and stored at −80°C until DNA extraction.

The primary endpoint was reduction of SA between weeks 0 and 8. Secondary endpoints were the increase in commensal organisms by weeks 4 and 8, reduction in SA (including MRSA) and, if present, *P. aeruginosa* by weeks 4 and 8, and tolerance to the spray. Exploratory endpoints included reduction of pain, wound closure by 50% or more during the 8 weeks, and recolonisation by pathogens from week 8 to 12 after cessation of spray.

### 2.2 Sample Collection and DNA Extraction

Genomic DNA was extracted using a Maxwell 16 LEV Blood DNA Kit (Promega, Madison, WI) implemented on a Maxwell 16 Instrument, following the manufacturer's instructions with minor modifications: lysozyme incubation (10 ng/μl lysozyme; Thermo Fisher Scientific, Waltham, MA) for 30 min at 37°C and bead beating (5 min at 30 Hz twice, 2 min rest in between) using a TissueLyser III (Qiagen, Germantown, MD). Homogenised samples were transferred to Maxwell cartridges for final DNA purification.

### 2.3 Full‐Length 16S rRNA Amplification and Sequencing

Genomic DNA was prepared for sequencing on a PacBio Revio sequencer using two‐stage PCR, similar to that described for Illumina amplicon library preparation [16]. In the first stage of PCR, DNA was PCR amplified with primers 27F and 1492R (TTTCTGTTGGTGCTGATATTGCAGRGTTYGATYMTGGCTCAG and ACTTGCCTGTCGCTCTATCTTC RGYTACCTTGTTACGACTT, respectively) using domain‐level bacterial primers as described by PacBio. The underlined regions, however, represent Oxford Nanopore (ONT) Universal sequences for tailing PCR primers' linker sequences. Reactions were performed in 10 μL reactions using repliQa HiFi ToughMix (Quantabio). Genomic DNA input was 4 μL per reaction. PCR conditions were 98°C for 2 min, followed by 24 cycles of 98°C for 10 s, 50°C for 2 s, and 68°C for 3 s. In the second stage of PCR, one microlitre of uncleaned PCR product from the first stage was used as a template and amplified with primers that contained PacBio Kinnex adapter sequences, combinatorial dual indices, and ONT linker sequences at the 3′ ends of the primers (e.g., Kinnex_Fwd and Kinnex_Rev, respectively: **CTACACGACGCTCTTCCGATC**TNNNNNNNNNNTTTCTGTTGGTGCTGATATTGC and **AAGCAGTGGTATCAACGCAGAG**NNNNNNNNNNACTTGCCTGTCGCTCTATCTTC). Kinnex adapters are bolded, combinatorial indices are indicated with “NNNNNNNNNN”, and ONT linkers are underlined. Second‐stage PCR cycling conditions were 98°C for 2 min, followed by 8 cycles of 98°C for 10 s, 60°C for 1 s, and 68°C for 1 s. PCR products from the 2nd stage were pooled, purified using a 0.5X Ampure cleanup, subjected to Kinnex library preparation, and loaded on a PacBio Revio instrument. DNA extraction and library preparation were performed at Rush University's Genomics and Microbiome Core Facility (GMCF). Kinnex library preparation and PacBio sequencing were performed at University of Illinois at Urbana‐Champaign's DNA Services Facility at the Roy J. Carver Biotechnology Center.

### 2.4 Basic Processing

Cutadapt v3.5 was used to trim forward and reverse primers from sequences. Reads were trimmed and filtered for quality using DADA2 v1.32.0, with the following changes from default parameters: acceptable lengths were set to between 100 (minLen) and 1600 bp (maxLen), minimum quality score (minQ) set to 3, maxEE of 2, and rm.phix as FALSE [17].

Error rates were estimated using PacBioErrfun, which is recommended for the analysis of PacBio circular consensus reads. To follow, reads were separated into ASVs using dada2's divisive partitioning machine learning approach. For both error modelling and divisive partitioning, band size was increased to 32 for sequence alignment in order to accommodate the increased rate of insertions and deletions in PacBio sequences [18]. Chimeric sequences were identified and removed using the *de novo* approach included within dada2, with minFoldParentAbundance increased to 3.5.

Resulting ASVs were assigned taxonomy using the default naive Bayesian classifier and SILVA v138.1 training set including species‐level classification [19]. To ensure investigation of relevant microbial communities, only taxa identified as bacteria at the kingdom level were kept. Additionally, any taxa unassigned at the phylum level were removed from the dataset. Decontam v1.24.0 was then applied to identify potential environmental contaminants using the environmental method blanks, with any above prevalence threshold 0.01 being removed from the ASV table [20]. Following decontamination, environmental samples were removed from the dataset. Lastly, any taxa prevalent in lower than 9% of samples were removed.

### 2.5 Statistical Analysis

Differences in clinical disease characteristics between timepoints were assessed using Student's t‐test. Correlation analyses were performed using Spearman's rank correlation. Missing longitudinal data was handled using the pairwise deletion method.

Samples in the cleaned ASV table were visually evaluated using Graphpad Prism v10.4, and R packages phyloseq v1.44.0 and ggplot2 v3.5.1 [21]. α‐diversity metrics, including observed species, Chao1, Shannon, and Simpson indices, were calculated by patient and timepoint. Using the R statistics package, a paired Wilcoxon signed‐rank test was performed to compare α‐diversity between timepoints across the patient cohort.

To evaluate overarching community differences, Bray‐Curtis distances were calculated between samples. This was followed by pairwise comparison of diversity across timepoints using pairWise Adonis v0.4 and R package vegan v2.6.8. To visualise community differences, PCoA plots were also generated.

Analysis of differentially abundant taxa between timepoints was performed using DeSeq2 v1.40.2 with Wald significance test and Benjamini‐Hochberg correction for multiple testing [22]. SA was assessed independently given its importance as a primary endpoint. Other significant ASVs were highlighted if their false discovery rate (BH‐FDR)‐adjusted *p*‐value was < 0.05 (*q*‐value).

## 3 Results

### 3.1 Patient Characteristics

Fifteen EB patients (11 DEB [7 RDEB and 4 DDEB]; 3 JEB; and 1 generalised EBS) were enrolled. Median age was 10 years with approximately equal males and females, 13.3% Black, and 26.7% Hispanic participants (Tables 1 and S1). Thirteen of 15 patients (87%) grew SA in culture; the remaining two had small wounds (≤ 1 cm^2^). Three patients (20%) grew MRSA and two *Pseudomonas aeruginosa* , in addition to MSSA (Table S1). The mean target wound size for all patients was 10 cm^2^ with median duration 6–12 weeks and mean pain score 4.4 at pre‐treatment baseline (Week 0).

**TABLE 1:** Summary of demographic and wound characteristics of enrolled subjects.

***N***		15				
**EB Subtype, *n* (%)**			**Week 0 Summary**			
	RDEB	7 (47)		Culture Results, *n* (%)		15
	DDEB	4 (27)			MSSA	10 (67)
	JEB	3 (20)			MRSA	3 (20)
	EBS	1 (6.7)			PA	2 (13)
**Age [yrs], median (range)**		10 (0.58–47)			Negative	2 (13)
**Sex, *n* (%)**				Wound size [cm^2^], mean (SD)^a^		10.0 (7.5)
	Male	7 (47)		Pain, mean (SD)^a^		4.4 (3.1)
	Female	8 (53)	**Week 8 Summary**			
**Race, *n* (%)**				Culture Results, *n*		12
	White	13 (87)			MSSA	6 (50)
	Black	2 (13)			MRSA	3 (25)
**Ethnicity, *n* (%)**					PA	1 (8.3)
	Hispanic	4 (27)			Negative	3 (25)
	Non‐Hispanic	11 (73)		Wound size [cm^2^], mean (SD)^a^		3.7 (7.7)
**BMI, mean (SD)**		17.9 (9.0)		Pain, mean (SD)^a^		3.1 (2.0)
**Wound Duration (%)**			Local Tolerability [Itch], mean (SD)^b^			1.1 (0.42)
	≤ 1 week	1 (6.7)	Local Tolerability [Burning/Stinging], mean (SD)^b^			2.1 (1.4)
	1–3 weeks	2 (13)	Satisfaction [Easy to Apply], mean (SD)^c^			4.9 (0.34)
	3–6 weeks	4 (27)	Satisfaction [Dries quickly], mean (SD)^c^			3.6 (1.3)
	6–12 weeks	1 (6.7)	Satisfaction [Feels good on skin], mean (SD)^c^			3.2 (0.90)
	> 12 weeks	7 (47)	Satisfaction [Preferred over prior topicals], mean (SD)^c^			3.7 (1.1)

Abbreviations: BMI, body mass index; DEB, dystrophic epidermolysis bullosa; EBS, epidermolysis bullosa simplex; JEB, junctional epidermolysis bullosa; MRSA, methicillin‐resistant *S. aureus* ; MSSA, methicillin‐sensitive *S. aureus* ; NA, not available; PA, *P. aeruginosa* ; SD, standard deviation.

^a^ Means calculated using only patients with both week 0 & 8 values.

^b^ Scored using Likert Scale 1–5, where 1 = None and 5 = Strong/Severe.

^c^ Scored using Likert Scale 1–5, where 1 = Strongly disagree and 5 = Strongly agree.

### 3.2 Change in Clinical Characteristics During Study Duration (Exploratory Endpoints)

For patients with complete longitudinal data, the mean wound size reduced from 10 cm^2^ at Week 0 to 6.7 cm^2^ at Week 4 (*p* = 0.25) and 3.7 cm^2^ (*p* = 0.048) at Week 8 (63.4% decrease). The mean pain score was 4.4 at Week 0, 3.8 at Week 4 (*p* = 0.50), and 3.4 at Week 8 (*p* = 0.40) (Tables 1 and S1). In general, the wound spray was well tolerated and product satisfaction scored highly. Local tolerability was excellent, with mean itch 1.1 (slight) and burning/stinging 2.1 (mild). Using the Likert scale for satisfaction, in which 1 = strongly disagree and 5 = strongly agree, mean scores for the spray were: easy to apply 4.9; dries quickly 3.6; feels good on the skin 3.2; and preferred to prior topicals 3.7 (Table 1).

### 3.3 Biodiversity and Microbial Communities

Full length 16S rRNA gene sequence data identified a total of 50 genera, 34 families, 20 orders, 8 classes, and 5 phyla. The environmental blank samples (*n* = 23), reagent, and PCR controls were negative for contamination. After sequencing, timepoints from two patients were excluded from analysis due to recent antibiotic usage; timepoints from an additional two patients were filtered out due to read counts below 1000.

From the remaining 89 samples across 14 patients, a total of 10 551 375 reads were obtained from PacBio sequencing, with an average of 118 555 reads per sample. The most abundant phyla were Firmicutes, Actinobacteriota, and Proteobacteria, while the most abundant species were SA, *Acinetobacter guillouiae* , and *Pseudomonas poae*.

**FIGURE 1:**
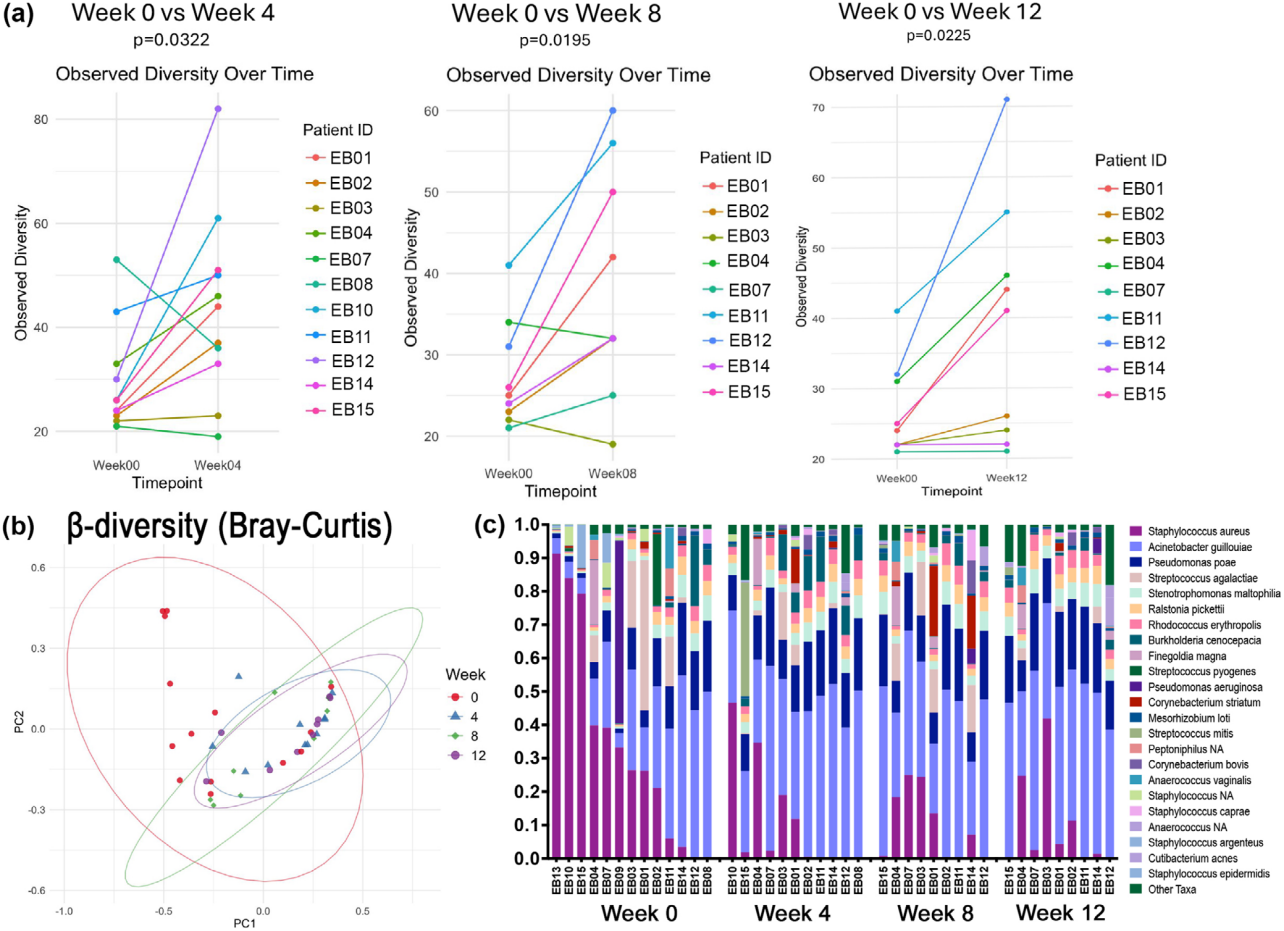
Increased bacterial diversity and altered bacterial community structures are observed in EB wounds treated with hypochlorous acid. (a) α‐diversity was higher at Weeks 4, 8, and 12 when compared to Week 0 (*p* < 0.05). (b) PCoA plots using the Bray–Curtis dissimilarity index of β‐diversity analyses show differential clustering of Week 0 wound samples compared to those of Week 4, 8 or 12 (*p* < 0.05). (c) Relative abundance (%) of the 20 most abundance species in EB wounds at Weeks 0 (before treatment); Weeks 4 and 8 (on treatment); and Week 12 (4 weeks after stopping treatment). Of note, the maroon colour on the vertical bars seen abundantly in the Week 0 microbiome is *S. aureus* , which is much decreased by as early as Week 4. The reduction persists in most specimens 4 weeks after the hypochlorous acid spray is discontinued.

At the species level, wound spray treatment led to an increase in α‐diversity, a metric of microbial diversity within samples. Significantly increased diversity was detectable by Week 4, the first sampled timepoint while on treatment, most pronounced at Week 8, and persistent through Week 12 (Figure 1). Notably, β‐diversity, a measure of dissimilarity between two microbial communities, revealed a difference between Week 0 and each later timepoint (all *p* < 0.05), but not between Weeks 4 and 8 or between Weeks 8 and 12.

### 3.4 SA in EB Wounds Decreases With Hypochlorous Acid Spray Use

**FIGURE 2:**
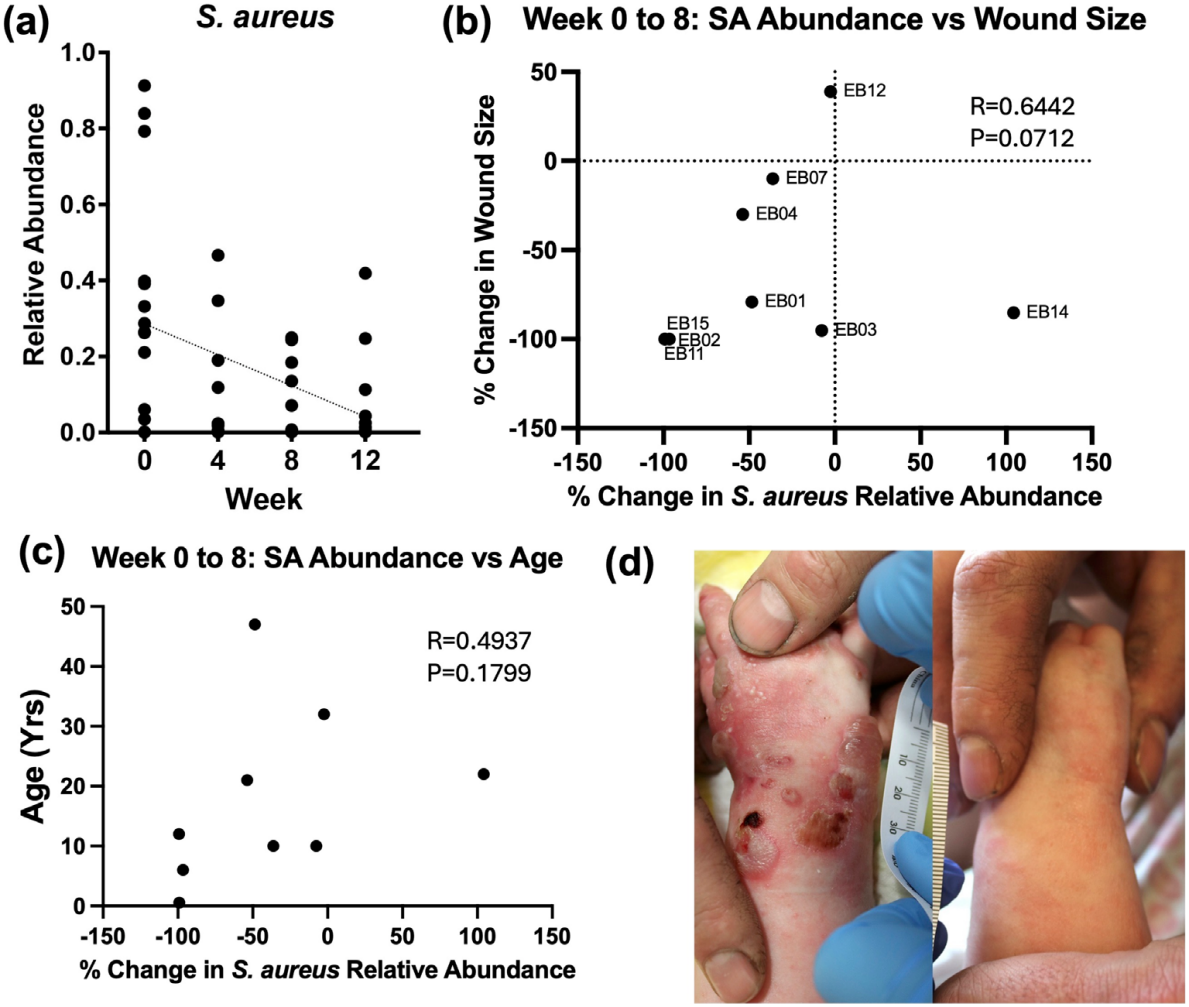
Changes in *S. aureus* abundance and wound size. (a, b) Relative abundance of *S. aureus* decreased throughout the study duration (8 weeks of treatment and 4 weeks after treatment discontinuation) and was associated with decreases in wound size from Week 0 to Week 8 (ρ = 0.64). (c) Younger age trended towards greater reduction in *S. aureus* at week 8 (ρ = 0.49). (d) Representative image of clinical improvement of wounds (EB15: 7‐month‐old at baseline with JEB) from week 0 (left) through week 8 (right).

The mean relative abundance of SA decreased from 34% at Week 0 to 11% at Week 4, 10% at Week 8 and, despite discontinuation of the spray at Week 8, 9.7% at Week 12. The primary endpoint of SA reduction from Week 0 to 8 was achieved (*p* = 0.01). The decrease in SA was also significant when comparing Week 0 to Week 4 (*p* = 0.0022) and even Week 0 to Week 12 (*p* = 0.02) (Figure 2). Notably, even patients who were culture‐positive for MRSA (mean 43% at Week 0, 17% at Week 4, and 17% at week 8) had decreased SA relative abundance. The decrease in SA relative abundance and decrease in wound size from Weeks 0 to 8 were strongly correlated (ρ = 0.64); but the *p*‐value missed significance (likely underpowered).

### 3.5 Younger Age Is Associated With Greater Reduction in SA

To better delineate factors that impact SA reduction, we also examined demographic and clinical characteristics, including age, sex, body mass index (BMI), and self‐reported race and ethnicity between Weeks 0 and 8. This analysis showed a moderate correlation between younger age and greater decrease in SA between Weeks 0 and 8 (ρ = 0.49, *p* = 0.18) (Figure 2c).

### 3.6 Changes in Other Microbiota Within EB Wounds With Hypochlorous Acid Use

Taxa‐by‐taxa analyses identified *S. argenteus* and *S. pyogenes* as other microbes that decreased from Week 0 to 4 or Week 0 to 8, respectively. On the other hand, commensal bacteria *Porphyromonas bennonis, Gemella haemolysans, Haemophilus haemolyticus*, and *Corynebacterium aurimucosum* were significantly higher, and *Anaerococcus hydrogenalis, Kocuria rhuzophilia, Neisseria cinerea, Staphylococcus caprae*, and *Anaerococcus vaginalis* trended higher (*p* < 0.05, FDR adjusted *p* > 0.05) at Week 4 when compared to Week 0. By Week 8, *Peptoniphilus coxii, Corynebacterium aurimucosum, Porphyromonas bennonis, and Staphylococcus caprae* were higher or trended higher compared to Week 0 (Table 2).

**TABLE 2:** Significantly altered bacterial species between Week 0 and Week 4 or 8.

	Bacterial species	LogFC	*p*	*q*
Week 4 *n* = 11	*Staphylococcus argenteus*	−13.590	< 0.0001	0.0006
	*Porphyromonas bennonis*	12.279	< 0.0001	0.0020
	*Gemella haemolysans*	11.806	0.0001	0.0029
	*Haemophilus haemolyticus*	11.126	0.0002	0.0055
	*Corynebacterium aurimucosum*	8.732	0.0013	0.0303
	*Staphylococcus aureus*	−3.109	0.0023	0.0428
	*Anaerococcus hydrogenalis*	7.940	0.0079	0.1277
	*Kocuria rhizophila*	7.421	0.0130	0.1842
	*Staphylococcus caprae*	5.363	0.0147	0.1842
	*Anaerococcus vaginalis*	4.551	0.0346	0.3703
	*Neisseria cinerea*	6.229	0.0360	0.3703
Week 8 *n* = 9	*Peptoniphilus coxii*	11.953	0.0001	0.0055
	*Corynebacterium aurimucosum*	10.380	0.0006	0.0227
	*Porphyromonas bennonis*	10.014	0.0009	0.0236
	*Streptococcus pyogenes*	−8.415	0.0046	0.0891
	*Staphylococcus caprae*	4.780	0.0054	0.0891
	*Staphylococcus aureus*	−2.332	0.0100	0.1364

Abbreviations: LogFC, log fold change; Negative FC, higher at week 0; *q*‐value, FDR adjusted *p*‐value.

## 4 Discussion

This pilot study demonstrates the potential efficacy, safety, and tolerability of an acid‐oxidising solution containing HOCl (APR‐TD011) in reducing SA relative abundance, increasing microbial diversity, and promoting healing in EB wounds. The findings extend existing research on microbial dysbiosis in EB and its role in wound pathophysiology, offering insights into therapeutic strategies.

### 4.1 SA and Wound Dysbiosis in EB

Using full‐length 16S rRNA gene amplicon sequencing, we observed a reduction in SA from 34% to 10% by Week 8, indicating the responsiveness of SA, including MRSA, to HOCl. This reduction aligns with the rapid action of APR‐TD011 in lowering pathogenic burden and supporting wound healing. Importantly, reduced SA abundance can mitigate SA serine protease V8‐mediated itch and inflammation, which are key contributors to delayed EB wound healing [23]. The observed correlation between reduced SA levels and wound healing underscores the significance of antimicrobial interventions in improving outcomes.

### 4.2 Microbial Diversity and Healing

EB wounds with reduced microbial diversity and dominance of pathogenic taxa often show poor outcomes. Our findings of increased microbial α‐diversity and decreased SA during treatment, persisting through Week 12, suggest a durable improvement in the wound microbiome and healing trajectory. This may reflect the restoration of protective commensal bacteria, which aid in immune modulation and suppression of pathogenic species.

### 4.3 Other Pathogenic Bacteria

Besides SA, reductions in *S. argenteus* and *S. pyogenes* were noted, highlighting APR‐TD011's broader antimicrobial efficacy. Though less studied in EB, these taxa are known for their virulence. For example, *S. argenteus* is a coagulase‐positive organism closely related to SA and produces similar toxins [24]. Two patients were culture‐positive for *P. aeruginosa* , which was not reflected in 16S data. Future work can explore specific bacterial roles in wound pathophysiology.

### 4.4 Wound Bed Preparation in EB and Choice of Disinfectant

Effective wound bed preparation is critical for EB healing and involves debridement, bacterial load reduction, and maintenance of a moist environment. APR‐TD011 facilitates these goals by controlling bioburden and promoting microbial diversity with a favourable safety profile. Its HOCl‐based composition, along with low pH and high redox potential, ensures broad‐spectrum antimicrobial activity while minimising tissue irritation. HOCl is preferred for EB wound care due to its biocompatibility, strong antimicrobial properties, and gentleness on tissues. In contrast, sodium hypochlorite solutions, though effective, pose a greater risk of irritation, particularly at higher concentrations [25]. Though no head‐to‐head trials exist for APR‐TD011 versus other agents in EB, HOCl's dual antimicrobial and anti‐inflammatory properties and its broad efficacy against MRSA and biofilms have been reported and may offer unique advantages [11, 12, 14].

### 4.5 Limitations and Future Directions

This study's small sample size and open‐label single‐arm design limit its generalizability. Variability in wound size, duration, and other confounders, such as nutrition and EB severity, may complicate interpretation. Natural healing cannot be fully excluded, but natural history studies suggest that RDEB wounds persist much longer than study duration [26]. Nevertheless, this study provides foundational data to guide future studies, including larger randomised controlled trials to validate these results, investigation of functional microbial changes, comparison against other wound etiologies (e.g., burns, leg ulcers, erosions in immune bullous disorders), and assessment of the impact of HOCl on inflammation, immune modulation, and long‐term outcomes, such as infection prevention and squamous cell carcinoma risk.

## 5 Conclusion

APR‐TD011 effectively reduces SA burden, enhances microbial diversity, and promotes wound healing in EB patients. By addressing microbial dysbiosis, this treatment offers a promising approach for reducing infection risks and improving EB‐related quality of life.

## Author Contributions

Conceptualization: Amy S. Paller, Lydia Rabbaa, Stephanie Rangel, and Xiaolong A. Zhou. Data Curation: Lydia Rabbaa, Amy S. Paller, Lynna Yang, and Michael B. Burns. Formal Analysis: Michael B. Burns, Elise Stagaman, Ziyou Ren, and Xiaolong A. Zhou. Funding Acquisition: Amy S. Paller. Investigation: Amy S. Paller, Lydia Rabbaa, and Lynna Yang. Methodology: Lydia Rabbaa, Amy S. Paller, Ziyou Ren, Stephanie Rangel, Stefan J. Green, and Lok Yiu Ashley Wu. Project administration: Lydia Rabbaa, Stephanie Rangel, and Amy S. Paller. Resources: Lydia Rabbaa, Amy S. Paller, Stephanie Rangel, Michael B. Burns, and Stefan J. Green. Software: Michael B. Burns, Ziyou Ren, and Xiaolong A. Zhou. Supervision: Amy S. Paller, Lydia Rabbaa, and Michael B. Burns. Validation: Michael B. Burns, Stefan J. Green, and Ziyou Ren. Visualisation: Xiaolong A. Zhou, and Michael B. Burns. Writing, original draft: Xiaolong A. Zhou, Amy S. Paller, and Lydia Rabbaa. All authors contributed to the article and approved the submitted version.

## Ethics Statement

Ethical approval was obtained from the Institutional Review Boards at Northwestern University and the Ann and Robert H. Lurie Children's Hospital of Chicago (IRB #2022–5523). Written informed consent or assent (if a minor ≥ 12 years old) was obtained from subjects or their legal guardians for their participation in this study.

## Conflicts of Interest

Amy S. Paller has served as an investigator for AbbVie, Applied Pharma Research, Dermavant, Eli Lilly, Incyte, Janssen, Krystal, Regeneron, Timber, and UCB, a consultant for AbbVie, Abeona, Apogee, Arcutis, Aslan, BioCryst, Boehringer‐Ingelheim, Bristol‐Myers‐Squibb, Dermavant, Incyte, Johnson and Johnson, Krystal Biotech, LEO, Mitsubishi Tanabe, Nektar, Primus, Procter and Gamble, Regeneron, Sanofi, Seanergy, TWI Biotech, and UCB, and on the data safety monitoring buoard for AbbVie, Abeona, and Galderma. Other authors have no conflicts of interest to disclose. Xiaolong A. Zhou has served as an investigator for Applied Pharm Research (this research).

## Supporting information


Table S1:

## Data Availability

The data that support the findings of this study are openly available in NCBI GEO at https://www.ncbi.nlm.nih.gov/geo/, reference number GSE288051.
